# Inter-epidemic Rift Valley fever virus infection incidence and risks for zoonotic spillover in northern Tanzania

**DOI:** 10.1371/journal.pntd.0010871

**Published:** 2022-10-28

**Authors:** William A. de Glanville, James M. Nyarobi, Tito Kibona, Jo E. B. Halliday, Kate M. Thomas, Kathryn J. Allan, Paul C. D. Johnson, Alicia Davis, Felix Lankester, John R. Claxton, Melinda K. Rostal, Ryan W. Carter, Rosanne M. F. de Jong, Matthew P. Rubach, John A. Crump, Blandina T. Mmbaga, Obed M. Nyasebwa, Emanuel S. Swai, Brian Willett, Sarah Cleaveland

**Affiliations:** 1 School of Biodiversity, One Health, and Veterinary Medicine, College of Medical, Veterinary and Life Sciences, University of Glasgow, Glasgow, United Kingdom; 2 University of Global Health Equity, Kigali, Rwanda; 3 Nelson Mandela African Institution of Science and Technology, Arusha, Tanzania; 4 Centre for International Health, University of Otago, Dunedin, New Zealand; 5 Kilimanjaro Clinical Research Institute, Moshi, United Republic of Tanzania; 6 School of Social and Political Sciences, University of Glasgow, Glasgow, United Kingdom; 7 Paul G. Allen School for Global Health, Washington State University, Pullman, Washington, United States of America; 8 Global Animal Health Tanzania, Arusha, Tanzania; 9 EcoHealth Alliance, New York, New York, United States of America; 10 Division of Infectious Diseases and International Health, Duke University Medical Center, Durham, North Carolina, United States of America; 11 Duke Global Health Institute, Duke University, Durham, North Carolina, United States of America; 12 Programme in Emerging Infectious Diseases, Duke-National University of Singapore, Singapore; 13 Kilimanjaro Christian Medical University College, Tumaini University, Moshi, Tanzania; 14 Ministry of Livestock and Fisheries, Dodoma, United Republic of Tanzania; 15 MRC University of Glasgow Centre for Virus Research, Glasgow, United Kingdom; University of Texas Medical Branch, UNITED STATES

## Abstract

Rift Valley fever virus (RVFV) is a mosquito-borne pathogen that has caused epidemics involving people and animals across Africa and the Arabian Peninsula. A number of studies have found evidence for the circulation of RVFV among livestock between these epidemics but the population-level incidence of infection during this inter-epidemic period (IEP) is rarely reported. General force of infection (FOI) models were applied to age-adjusted cross-sectional serological data to reconstruct the annual FOI and population-level incidence of RVFV infection among cattle, goats, and sheep in northern Tanzania from 2009 through 2015, a period without reported Rift Valley fever (RVF) cases in people or animals. To evaluate the potential for zoonotic RVFV spillover during this period, the relationship between village-level livestock RVFV FOI and human RVFV seropositivity was quantified using multi-level logistic regression. The predicted average annual incidence was 72 (95% Credible Interval [CrI] 63, 81) RVFV infections per 10,000 animals and 96 (95% CrI 81, 113), 79 (95% CrI 62, 98), and 39 (95% CrI 28, 52) per 10,000 cattle, sheep, and goats, respectively. There was variation in transmission intensity between study villages, with the highest estimated village-level FOI 2.49% (95% CrI 1.89, 3.23) and the lowest 0.12% (95% CrI 0.02, 0.43). The human RVFV seroprevalence was 8.2% (95% Confidence Interval 6.2, 10.9). Human seropositivity was strongly associated with the village-level FOI in livestock, with the odds of seropositivity in an individual person increasing by around 1.2 times (95% CrI 1.1, 1.3) for each additional annual RVFV seroconversion per 1,000 animals. A history of raw milk consumption was also positively associated with human seropositivity. RVFV has circulated at apparently low levels among livestock in northern Tanzania in the period since the last reported epidemic. Although our data do not allow us to confirm human RVFV infections during the IEP, a strong association between human seropositivity and the FOI in cattle, goats, and sheep supports the hypothesis that RVFV circulation among livestock during the IEP poses a risk for undetected zoonotic spillover in northern Tanzania. We provide further evidence for the likely role of raw milk consumption in RVFV transmission from animals to people.

## Author summary

Rift Valley fever outbreaks are reported in Tanzania every 10 to 15 years. Human and animal cases are not typically reported in the period between outbreaks, but the RVF virus (RVFV) is known to circulate among livestock during these periods. The last officially reported outbreak of RVF in Tanzania was in 2008. The incidence of RVFV infection among livestock in the period since this large epidemic in the country is not known. We used a general force of infection model to quantify the annual incidence of RVFV infection among livestock in northern Tanzania between 2009 and 2015, a period without reported RVF cases in people or animals. Using a sample of over 9,000 animals from 43 villages across northern Tanzania, we estimated a moderately low average annual incidence of 96, 39, and 79 RVFV infections per 10,000 cattle, goats, and sheep, respectively. There was considerable heterogeneity in the intensity of transmission between villages, highlighting important spatial differences in inter-epidemic RVFV transmission among livestock in this region. We also found that human seropositivity was strongly positively associated with the village-level force of RVFV infection among livestock. All people with seropositive results in this study were born before 2008. We therefore cannot be certain that these people were infected in the period since the last outbreak. However, the very strong positive association we find between inter-epidemic circulation of RVFV among livestock and human seropositivity provides support to the hypothesis that zoonotic RVFV spillover may be occuring during the period between large-scale outbreaks in northern Tanzania. Further studies are required to confirm this. We also found a strong positive association between a history of raw milk consumption and human RVFV seropositivity, providing further evidence for the importance of milk as a likely route for zoonotic RVFV transmission.

## Introduction

Rift Valley fever (RVF) is a mosquito-borne disease caused by the Rift Valley fever virus (RVFV). The epidemiology of RVF in endemic areas is characterised by infrequent epidemics triggered by the emergence of large numbers of flood-water mosquitoes during periods of unusually heavy rainfall [1]. In East Africa, these mosquito emergence events, and RVF epidemics, are typically associated with the warm phase of the El Niño-Southern Oscillation (ENSO) [2,3]. Epidemics are followed by an inter-epidemic period (IEP) in which clinical disease in people and animals is generally not reported. In Tanzania, RVF epidemics have been reported every 10 to 15 years since the 1930s, with the last officially reported outbreak occurring in 2007 and 2008 and involving more than 130,000 livestock infections and at least 264 human illnesses with 109 deaths [4,5]. A much smaller-scale RVF outbreak that was identified on the basis of retrospective RVFV testing of livestock abortion samples collected in 2018 was also recently described [6,7].

Identifying the mechanisms that allow RVFV maintanence during the IEP is an active area of research [8–10]. Early hypotheses for maintenance were centred around trans-ovarial transmission of the virus by some *Aedes* spp. mosquito species and the survival of infected, dessication-resistant eggs for long periods in the soil of previously flooded areas [2]. Such theories suggested viral dormancy and an absence of sustained transmission among livestock or to people outside of epidemics. However, a growing number of serological surveys in RVFV endemic areas, including Tanzania [11–15], have identified human and animal infections during the IEP. In general, this evidence comes from detection of seroconversion among people or animals that were born in the period since the last locally reported disease outbreak [16,17], or the detection of IgM antibodies suggesting recent infection [15]. Although the transmission of RVFV during the IEP in endemic areas is now well recognised [1], many questions remain about the dynamics of RVFV transmission outside of epidemics, including the population-level infection incidence during periods without reported human and animal cases [1,18].

Cohort studies and sentinel-based surveillance have been advocated for monitoring RVFV transmission dynamics during the IEP [19,20]. However, for low levels of pathogen transmission, as are expected for RVFV during the IEP, these activities would typically need to be very large or be restricted to known high risk areas in order to detect infections. In comparison to the large number of reported cross-sectional RVFV sero-surveys [21], relatively few cohort-based studies of livestock RVFV infection during the IEP have been reported [16,22–25]. An alternative approach to estimate the levels of transmission of RVFV over defined periods, such as during the IEP, is to fit general force of infection (FOI) models to age-specific sero-survey data generated from cross-sectional studies. Such models have been used widely in infectious disease epidemiology [26], and have been applied to reconstruct historical RVFV transmission among livestock in Madagascar [27] and Cameroon [28]. Assuming the development of long lasting antibodies following infection, which is a reasonable assumption for infection with RVFV [29], these catalytic models allow reconstruction of the force of RVFV infection on the basis of age-specific antibody prevalence [30]. While the detection of antibodies to RVFV among unvaccinated animals born during the IEP can provide good evidence for RVFV transmission during the IEP, estimation of FOI from age-specific seroprevalence data enables quantification of the expected levels of transmission over specific time periods (such as per year). Not only can this provide richer data on RVFV transmission dynamics during the IEP [27,28], it can also provide a baseline for the expected level of RVFV infection per unit time that can inform surveillance and disease planning activities. The estimation of FOI of an endemic disease can also have particular advantages over population-level seroprevalence estimates, which will tend to reflect the age-composition, and therefore cumulative exposure time, of a population. [30]. This advantage may be particularly relevant for serosurvey data for multi-host pathogens, such as RVFV, from livestock keeping communities such as those in East Africa in which a diversity of species (including humans) with wide-ranging life-spans may be included in data analysis.

We used age-specific serological data generated from two large cross-sectional sero-surveys conducted in three administrative regions of northern Tanzania to reconstruct the average annual FOI and population-level incidence of RVFV infection in cattle, goats, and sheep from 2009 through 2015, a period without reported RVF cases in animals or people. To test the hypothesis that virus transmission among livestock during this IEP could also be associated with undetected human infections, we assessed the association between estimates of village-level livestock RVFV FOI and the risk of RVFV seropositivity in people living in the same villages. Potential mediators of viral spillover from livestock to people, including an individual person’s history of animal contact and animal source food consumption, were then explored.

## Materials and methods

### Ethics statement

The study protocols, questionnaires, and consent documents were approved by the Kilimanjaro Christian Medical Centre Ethics and Review Committee (KCMC CRERC Ref. 832 and 535) and National Institute of Medical Research Ethics Review Committee (NIMR ERC Ref. 2028 and 1522), the University of Otago Ethics Committee (Ref. H17/069), University of Glasgow Medical, Veterinary and Life Sciences Ethics Committee (Ref. 200140152), and the Institutional Review Board for Clinical Investigations of Duke University Health System (Ref. 000373560). Permission to carry out the study in Tanzania was provided by the Tanzania Commission for Science and Technology (Ref. 2014-244-ER-2005-141). Written informed consent or assent for sample collection and questionnaire administration was collected from all participants in all studies. Written informed consent was also obtained from the parent or legal guardian for children. Permission to publish this work was granted by the Director of Veterinary Services, Tanzania and by the publication review committee at NIMR.

### Serological surveys

Sera were available from two cross-sectional sero-surveys. The first comprised samples collected as part of the ‘Impact and Social Ecology of Bacterial Zoonoses in Tanzania’ (BacZoo) study [31]. The second comprised samples collected as part of the ‘Social, Economic, and Environmental Drivers of Zoonotic Disease in Tanzania’ (SEEDZ) study [32].

The methods used in the BacZoo study have been described in detail elsewhere [31,33]. Briefly, administrative wards in Arusha and Kilimanjaro Regions were first classified into the three main agro-ecological systems thought to be present in the area (peri-urban, agro-pastoral, and pastoral) by local experts (typically the district veterinary or livestock officer) [29]. Seven, seven, and six wards were then randomly selected from those classified as peri-urban, agro-pastoral, and pastoral, respectively, and a single village randomly selected from among all villages in each selected ward. Data collection was conducted in in six villages in two districts in Arusha Region and 14 villages in five districts in Kilimanjaro Region. A census of all livestock keeping households was completed in these villages, and between 4 and 11 households randomly selected from this census for recruitment. Within recruited households, up to 15 of each of cattle, goats, and sheep were selected for blood sampling. Where more than 15 of a particular species was owned by a household, a preference for sampling was given to adult female animals since the BacZoo study was focussed on a range of questions around livestock-associated zoonotic disease. All assenting or consenting human members of the household that were normally resident and aged five years or greater also had a blood sample drawn. A household-level questionnaire focusing on demographics and livestock management practices was conducted with the household head. An individual-level questionnaire focusing on animal contact and animal-source food consumption was conducted with each person sampled. All data were collected from September 2013 through March 2015.

Methods for the SEEDZ study have also been described in detail elsewhere [32,34,35]. Initially, livestock and human samples were collected from three randomly selected villages in randomly selected pastoral, agropastoral and peri-urban wards in two districts of Arusha Region following all protocols from the BacZoo study (described above). An additional 20 villages were then selected from across Arusha (10 villages) and Manyara (10 villages) Regions. These 20 villages were selected using a generalised random tessellation stratified sampling approach to provide a spatially balanced random sample [32]. Following the approach used by the BacZoo study, stratification was performed based on agro-ecological system, with 11 villages selected from within those classified as pastoral and nine villages from those classified as non-pastoral by local experts (typically the district veterinary or livestock officer). Villages in wards classified as urban during the 2012 Tanzanian census were excluded. Villages already sampled through BacZoo were also excluded. Livestock sampling in each of these 20 villages was conducted at two to three ‘central points’ to which livestock keepers were invited to bring their animals. Households were registered on arrival and 10 were selected at random from the registry (with a total of 20 to 30 per village) for sampling. Up to twelve of each of cattle, goats, and sheep were sampled from each of the selected households. Where more than 12 animals of each species were owned by a household, selection was limited to five immature animals (less than 18 months for cattle and less than 12 months for sheep and goats) with the remaining seven from adult animals. Animals less than six months of age were excluded. Participating households were visited within one week and a household questionnaire focusing on demographics and livestock management practices conducted with the household head. In a random selection of households, whole blood was collected from all assenting or consenting household members normally resident and aged five years or older. The same individual-level questionnaire as used in the BacZoo study was also administered. Data were collected from January through December, 2016.

Whole blood samples from livestock and humans were allowed to clot before centrifugation and serum extraction. An aliquot of each serum sample was heat treated at 56°C for 120 minutes before shipment under license (TARP(S) 2016/49) to the University of Glasgow, UK, for serological testing.

### Serological testing

All livestock and human sera from the BacZoo study and all cattle and human sera from the SEEDZ study were tested for presence of RVFV IgG using a commercial multi-species competitive ELISA (ID Screen, IDVet, Paris, France). This test has been estimated to have a diagnostic sensitivity of 85% (95% credible interval (CrI) 65.5, 99.1) and a specificity of 99% (95% CrI 97.1, 99.8) for the detection of RVFV IgG using cattle samples from West Africa [36]. Testing of the SEEDZ sheep and goat sera involved an additional step. For these samples, sera were first screened using an in-house nucleocapsid-based ELISA developed at the University of Glasgow [37]. Positives on this in-house ELISA were then confirmed using the multi-species competitive ELISA described above. Only sheep and goat samples positive on the multi-species competitive ELISA assay were considered seropositive. Percent positive agreement between the in-house assay performed on SEEDZ sheep and goat samples and the multi-species competitive ELISA assay was assessed using a random subset of in-house assay negatives and all in-house assay positives (see S1 File for further details).

The multi-species competitive ELISA used in this study was marketed for use in livestock. Several other research studies have used this assay for the assessment of human seropositivity [38,39]. A random subset of human samples that were multi-species competitive ELISA positive were re-tested using the plaque reduction neutralisation test (PRNT), the reference serological assay for RVF [40] (see S1 File for further details).

### Estimating FOI and annual incidence of livestock RVFV infection

We estimated the FOI of RVFV among cattle, goats, and sheep born from 2009 through 2015 using the merged dataset from both serosurveys. All animals included in the analysis were therefore born after the 2007/2008 RVF outbreak and before the most recent 2018 outbreak. The FOI (λ) is linked to the age-specific probability of exposure (*P*_*a*_) within a catalytic framework as [30]:

$$P_{a}=1-exp[-\lambda a]$$ (1)

Where age (*a*) is measured in years, the FOI represents the proportion of previously uninfected (antibody negative) animals that are exposed (becoming antibody positive) to infection per year. This interpretation assumes a constant rate of infection in the population, no specific loss from the population due to RVFV infection (e.g., through disease mortality), no seroreversion following infection, and no age dependency with respect to exposure to infection or the likelihood of developing antibodies post infection [30].

Rearranging Eq 1 to derive the FOI, and then taking natural logs represents the complementary log-log link function for the age-specific probability of exposure [30]. Hence, the FOI can also be directly derived from seroprevalence data using a binomial generalised linear model (GLM) with a clog-log link and the natural log of age as an offset. We used this approach to estimate the average annual FOI for the all-species dataset and seperately for cattle, goats, and sheep. To explore between-village variation in FOI, we extended this GLM to include a random effect at the village-level (i.e., a generalised linear mixed model [GLMM]). Our primary motivation was to identify between village variation in FOI rather than to explain it, and no fixed effects were included in this null model. Evidence for spatial autocorrelation in village-level infection risk (residual spatial correlation, RSA) was assessed on the basis of the Moran’s I statistic using residuals from the all-species model.

Since the FOI relates to infection among previously uninfected individuals, but the population of livestock in northern Tanzania comprises both seropositive and seronegative animals, we derived an estimate of the average annual population-level incidence (*I*) per 10,000 animals in the study area from average annual FOI and the population-level prevalence (*P*) as [41]:

I=λ(1−P)×10000 (2)

### Estimating animal age

Exact animal age is often unknown in this setting, where written animal records are rarely kept, herd sizes can be large, and animals are frequently introduced through purchase, borrowing, and gifting [32]. We therefore assigned an approximate age to each animal on the basis of its dentition. Age for animals with a mix of temporary and permanent (adult) teeth was assigned as the mid-point between the lowest expected age of eruption of a pair of permanent incisors and the upper expected age of eruption of the next pair of permanent incisors [42–44]. Animals with four pairs of permanent incisors (a ‘full mouth’) can be further categorised into ‘unworn’ and ‘worn,’ with a worn set of permanent incisors indicating advanced age. We used farmer estimates of age for cattle, goats, and sheep with ‘full and unworn’ and ‘full and worn’ incisors to derive an average age for animals in these two categories. Animal age categories are described further in the S1 File.

### Identification of predictors and mediators of RVFV exposure risk in humans

Logistic regression was used to identify predictors and mediators of human seropositivity to RVFV. The analysis proceeded in four stages. First, a null model containing only a random effect at the village level was specified. Second, the village-level residual FOI of RVFV infection (derived from the GLMM described in section 2.3.1) was included as a potential predictor of human RVFV seropositivity together with an individual’s age and sex. Third, the effect of potential mediators of village-level livestock residual FOI was assessed. Mediators were individual-level variables that were expected to act in the causal pathway between livestock and human RVFV infection [45]. Comparison of the co-efficient for village-level FOI with and without these mediators was taken to provide an indication of the extent to which included variables could explain the association between incident livestock infections and human seropositivity (i.e., to act as mediators in zoonotic spillover) [45]. We included the following mediators: consumption of raw milk in the past month; consumption of raw blood in the past month; milking livestock in the past month; herding livestock in the past month; contact with livestock carcasses in the past 12 months; contact with livestock abortus in the past 12 months; involvement in animal birthing in the past 12 months; and involvement in animal slaughter in the past 12 months. Recall period (one month or 12 months) was chosen based on the expected frequency of these activities in this setting. We did not have data on exposure to mosquitoes, which could also be expected to act as a mediator between livestock and human RVFV infection. Fourth and finally, and given the number of potentially correlated predictors, as well as the expectation of a small number of individuals with RVFV exposure, we compared co-efficients from a full model containing all variables with those from a model selected using penalised regression using the least absolute shrinkage and selection operator (LASSO) [46].

The relative contribution of village-level FOI in explaining between-village variation in human RVFV seropositivity was quantified by measuring the proportional change in variance (PCV) when comparing the null logistic regression model with each of the models that included village-level FOI as a fixed effect [47].

### Model specification and evaluation

All models were formulated within a Bayesian framework in JAGS via the R package, *R2jags* [48] in R version 3.6.1. The R code used to specify the likelihood and priors for each FOI model in JAGS is given in the S1 File. Convergence for parameter estimates was assessed by visual examination of three MCMC chains after a minimum burn-in of 50,000 and at least 100,000 iterations. We used a double exponential prior on fixed effect co-efficients and weakly informative normal priors for random effects for the LASSO model of human seropositivity [49]. For all other models, weakly informative normal priors were used for both fixed and random effects. Precision for random effects was defined using a wide uniform hyperprior. The Moran’s I statistic for residuals from the livestock GLMM was derived using the *sdpep* package [50] in R.

### Sensitivity analysis of the impacts of test misclassification on FOI estimation

An important assumption in our estimation of FOI is that animal seropositivity reflects prior infection with RVFV. To test the impacts of this assumption, we assessed the consequences of false positive test misclassification on estimates of FOI for the all-species model *Oa*_*a*_. We used a range of 90 to 100% as realistic but conservative lower and upper bounds for diagnostic specificity of the multi-species competitive ELISA based on uncertainty estimates from published test performance evaluations [36,51]. Given the low expected overall prevalence of RVFV infection in this population, we were generally less concerned about false negative results and performed an initial analysis with diagnostic sensitivity fixed at 100%. We repeated this with diagnostic sensitivity fixed at 85% as the reported sensitivity of the commercial cELISA assay among cattle in West Africa [36].

In this sensitivity analysis, the data were considered to represent the age-specific probability of testing positive on the basis of the multi-species competitive ELISA (*PA*_*a*_), which can be linked to the ‘true’ age-specific probability of infection (*P*_*a*_, from Eq 1) through the following equation [52]:

$${PA}_{a}=P_{a}\times Sensitivity+(1-P_{a})\times (1-Specificity)$$ (3)

## Results

### Livestock RVFV seroprevalence

**Fig 1:**
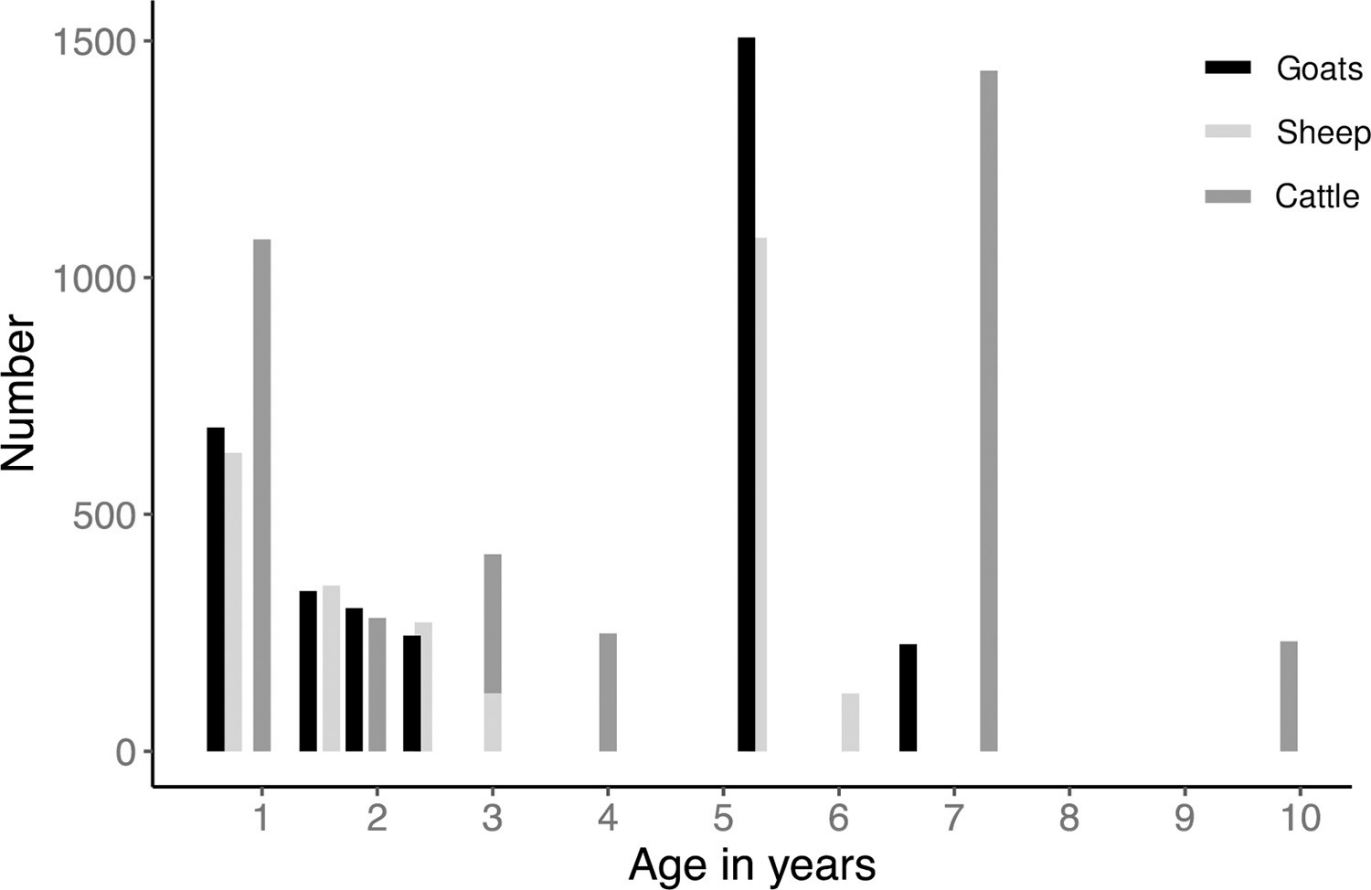
Number of cattle, goats, and sheep sampled by age from two cross-sectional serosurveys conducted in 2013 through 2016 in Arusha, Manyara and Kilimanjaro Regions, Tanzania.

A total of 9,476 livestock sera samples were collected in the two serosurveys, of which 3,582 (38%) were cattle, 3,303 (35%) were goats, and 2,584 (27%) were sheep. Samples came from 563 households in 43 villages across Arusha, Kilimanjaro and Manyara Regions. The majority of animals of each species were classed as being in the unworn full mouth category, representing an age of 7.3 years in cattle, 5.2 years in goats, and 5.3 years in sheep (Fig 1). The average (median) age for cattle, goats, and sheep was 4.6 (4.0), 3.4 (5.2), 3.3 (3.0) years, respectively. The overall seroprevalence of RVFV exposure was 2.8% (n = 268 seropositive animals, 95% Confidence Interval [CI] 2.5, 3.2). Overall seroprevalence was highest in cattle (4.4%, n = 156, 95% CI 3.7, 5.1), followed by sheep (2.6%, n = 67, 95% CI 2.0, 3.3), and goats (1.4%, n = 45, 95% CI 1.0, 1.8).

The observed percent positive agreement when comparing the in-house assay used for SEEDZ sheep and goat samples with the commercial cELISA was 98% (see S1 File).

### Livestock RVFV FOI and population-level infection incidence

**Fig 2:**
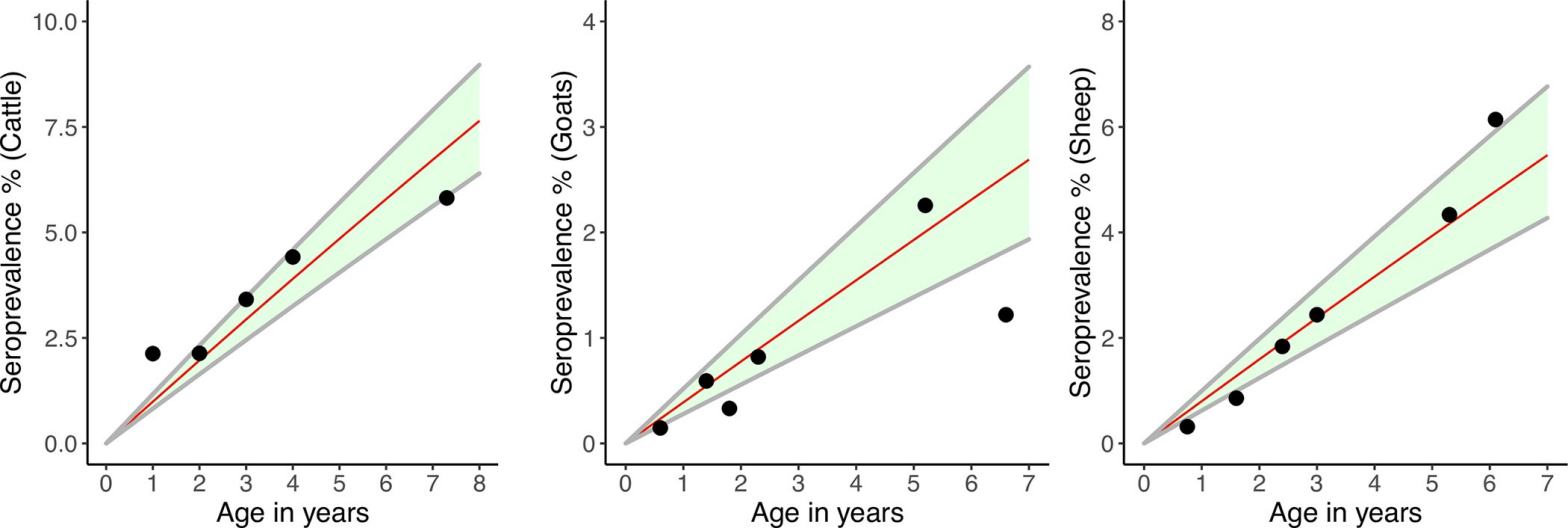
Predicted RVFV seroprevalence (red line) and 95% credible intervals (grey line) by age on the basis of catalytic models for cattle, goats and sheep using data collected in northern Tanzania from 2013 through 2016. Observed seroprevalence by age for each species is overlaid as black circles.

The annual predicted FOI of RVFV infection for all livestock species was 0.74% (95% Credible Interval [CrI] 0. 65, 0.84). This was 0.99% (95% CrI 0.82, 1.20), 0.82% (95% CrI 0.64, 1.02), and 0.40% (95% CrI 0.29, 0.53) among cattle, sheep, and goats, respectively. Predicted age-specific seroprevalence on the basis of the estimated FOI of infection for each species is given in Fig 2. The population-level annual incidence of RVFV infection among livestock was estimated to be 72 (95% CrI 63, 81) per 10,000 animals. This was 96 (95% CrI 81, 113), 79 (95% CrI 62, 98), and 39 (95% CrI 28, 52) per 10,000 cattle, sheep, and goats, respectively.

The impact of test misclassification on RVFV FOI for all livestock is shown in Table 1. Estimates were strongly influenced by diagnostic specificity. The expected annual FOI fell by around 30% if the diagnostic specificity was reduced from 100 to 99%, and to essentially zero when diagnostic specificity was 94% or below (Table 1, values below 94% not shown). Expected annual FOI was slightly higher with incorporation of the expected 85% diagnostic sensitivity of the cELISA assay, although there was substantial overlap in 95% credibile intervals compared with estimates derived on the assumption of 100% diagnostic sensitivity.

**Table 1:** Effect of variation in diagnostic specificity on the estimate of average annual force of infection (FOI) of Rift Valley fever virus infection in Tanzanian cattle, goats, and sheep between 2009 and 2015.

Specificity (%)	FOI (% (95% CrI)): 100% sensitivity	FOI (% (95% CrI)): 85% sensitivity
100	0.74 (0.65, 0.84)	0.87 (0.76, 0.99)
99	0.50 (0.41, 0.59)	0.58 (0.48, 0.70)
98	0.30 (0.21, 0.39)	0.35 (0.25, 0.46)
97	0.11 (0.05, 0.21)	0.13 (0.04, 0.24)
96	0.02 (0.01, 0.07)	0.03 (0.08, 0.09)
95	0.01 (0.00, 0.04)	0.01 (0.00, 0.05)
94	0.008 (0.00, 0.03)	0.009 (0.00, 0.03)

### Village-level variation in RVFV FOI in livestock in northern Tanzania

**Fig 3:**
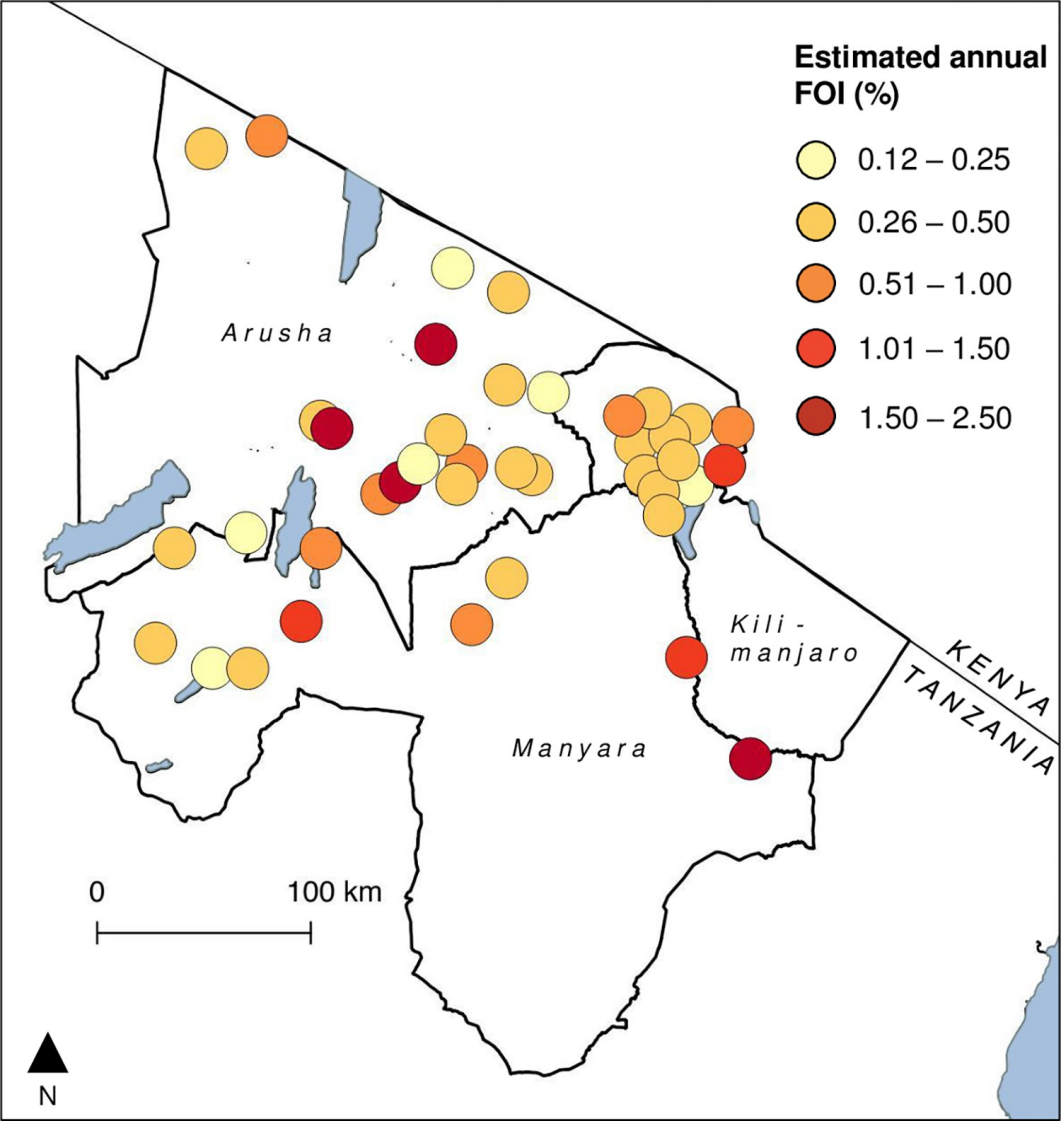
Variation in the village-level annual predicted force of infection (FOI) of Rift Valley fever virus among livestock between 2009 and 2015 in Arusha, Manyara and Kilimanjaro Regions of northern Tanzania. Map created using QGIS version 3.22.1. Base layer from GADM (https:gadm.org/).

The village-level annual FOI ranged between 0.12% (95% CrI 0.02, 0.43) and 2.49% (95% CrI 1.89, 3.23) (Fig 3). These figures can be interpreted as between approximately 0.1 and 2.5% of previously uninfected cattle, goats, and sheep in study villages being infected with RVFV on an annual basis in the years between 2009 and 2015. There was no evidence for RSA in village-level FOI residuals (Moran’s I statistic = 0.07, p-value = 0.14).

### Human RVFV seroprevalence in northern Tanzania

Serological samples were available from 558 people from 234 households. Of these, 46 (8.2%, 95% CI 6.2, 10.9) were RVFV seropositive on the basis of the multi-species competitive ELISA. A total of 549 (98%) had an available date of birth or age category data. No seropositive persons were identified among the 33 (6%) of these 549 participants recorded as aged less than 12 years (and therefore born in the period since the 2007/2008 RVF epidemic in Tanzania). Table 2 summarises the characteristics of all study participants and the number and proportion with each characteristic that were seropositive. Twenty five (4%) individuals had missingness in age, sex and/or any of the mediators, and were excluded from all of the logistic regression models.

**Table 2:** Characteristics of human study subjects in relation to RVFV serostatus in northern Tanzania using samples collected from 2013 to 2016.

Characteristic		Total number (%)	Number (%) RVFV seropositive
Age	5–18 years	78 (14.4)	3 (3.8)
	19–34 years	144 (26.6)	18 (12.5)
	35–54 years	189 (34.9)	14 (7.4)
	> 55 years	131 (24.1)	10 (7.6)
Sex	Female	291 (53.0)	19 (6.5)
	Male	258 (47.0)	27 (18.2)
Animal slaughter in past 12 months	No	304 (55.4)	13 (4.6)
	Yes	245 (44.6)	32 (13.1)
Birthing animals in past 12 months	No	339 (62.0)	15 (4.4)
	Yes	208 (38.0)	31 (14.9)
Blood consumption in past month	No	486 (88.5)	37 (7.6)
	Yes	63 (11.5)	9 (14.3)
Carcass contact in past 12 months	No	383 (69.8)	25 (6.5)
	Yes	166 (30.2)	21 (12.7)
Contact with abortus in past 12 months	No	481 (87.6)	34 (7.1)
	Yes	68 (12.4)	12 (17.6)
Herding animals in past month	No	278 (50.8)	16 (5.8)
	Yes	269 (49.2)	30 (11.2)
Milking animals in past month	No	342 (62.4)	29 (8.5)
	Yes	206 (37.6)	17 (8.3)
Raw milk consumption in past month	No	452 (82.3)	22 (4.9)
	Yes	97 (17.7)	24 (24.7)

Of the 46 ELISA positive individuals, a random selection of 26 (57%) were tested with the PRNT. All were positive on the basis of this second test. A comparison between ELISA and PRNT results is given in the S1 File.

### Predictors and mediators of RVFV exposure risk in humans

Human seropositivity was strongly positively associated with the inter-epidemic village-level RVFV FOI in livestock (Table 3), with the risk of seropositivity increasing by around 1.2 times (95% CrI 1.1, 1.3) for each additional average annual seroconversion per 1,000 animals (estimated by unscaling the odds ratio for model including predictors of human RVFV seropositivity shown in Table 3). There was no evidence for a relationship between linear age or sex and human seropositivity. The model with predictors of human seropositivity (FOI, age, and sex) explained over half of the between-village variation in human serostatus (a PCV of 57%, Table 3). A history of raw milk consumption in the past 30 days was strongly positively associated with human RVFV seropositivity in the model with all mediators (Odds Ratio (OR) 3.67 (95% Cr I 1.58, 8.14), Table 3). There was relatively weaker evidence (i.e., the lower bound of the 95% credible interval was close to a value of one) for a positive relationship between contact with an animal abortus and RVFV seropositivity (OR 2.68 (95% Cr I 1.02, 7.27), Table 3). The inclusion of these and all other individual-level mediators of RVF risk had only a moderate mediating effect on the village-level livestock FOI (i.e., the odds ratio for the effect of village-level livestock FOI with these mediators was only moderately smaller than the odds ratio without them). However, this model containing all predictors and potential mediators explained a substantial proportion of the total village-level variation in human serostatus (a PCV of 74%). A strong positive effect of a history of raw milk consumption on the odds of human seropositivity persisted in the model selected using variable selection and regularisation based on the LASSO, albeit with weaker evidence for an association (Table 3). A strong positive relationship with RVFV FOI among livestock during the IEP also persisted.

**Table 3:** Parameter estimates from logistic regression analysis of Rift Valley fever virus seropositivity in humans in northern Tanzania using 533 samples collected from 2013 through 2016.

	Null model of RVF seropositivity	Predictors of RVF seropositivity	Predictors and mediators of RVF seropositivity	Variables selected by LASSO
**Fixed effects**	-	Odds ratio (95% CrI)	Odds ratio (95% CrI)	Odds ratio (95% CrI)
Livestock FOI^1^^a^	-	**2.73 (1.61, 4.90)** *	**2.31 (1.47, 4.02)** *	**1.96 (1.19, 3.15)** *
Age^1^^,^^2^	-	1.09 (0.74, 1.56)	1.21 (0.79, 1.87)	1.10 (0.81, 1.57)
Sex (male)	-	1.90 (0.93, 3.89)	0.90 (0.30, 2.77)	1.10 (0.61, 2.06)
Birthing animals	-	-	1.83 (0.74, 4.64)	1.48 (0.84, 3.17)
Blood consumption	-	-	0.58 (0.21, 1.55)	0.88 (0.43, 1.53)
Carcass contact	-	-	1.29 (0.54, 3.03)	1.17 (0.70, 2.25)
Contact with abortus	-	-	**2.68 (1.02, 7.27)** *	1.60 (0.85, 3.93)
Herding animals	-	-	1.60 (0.65, 4.04)	1.30 (0.77, 2.59)
Milking animals	-	-	0.74 (0.25, 2.09)	0.91 (0.48, 1.55)
Raw milk consumption	-	-	3.67 (1.58, 8.14)*	2.10 (1.01–4.83)*
Slaughter animals	-	-	1.76 (0.79, 4.28)	1.42 (0.84, 2.83)
**Variance of random effects**				
$\sigma_{village}^{2}$ ^#^	2.9 (0.9, 7.3)	1.3 (0.2, 3.6)	0.8 (0.0, 2.7)	0.8 (0.0, 2.6)
PCV (%)^b^	*Reference*	57%	74%	74%

^1^Scaled to have mean of zero and standard deviation of one

^2^ Four categories of approximately 20 years (0–18, 19–34; 35–54; 55 and above) treated as a continuous variable

^#^ 95% credible intervals given in parentheses

* 95% credible intervals do not include 1

^a^ Force of infection (FOI)

^b^ Proportional change in variance (PCV).

## Discussion

Our study provides further evidence for the circulation of RVFV in cattle, goats, and sheep in Tanzania over a period in which no cases of RVF were reported in either humans or animals. The average annual incidence of livestock RVFV infection in northern Tanzania was moderately low but we observed substantial variation in the FOI between villages, suggesting important spatial heterogeneity in transmission intensity and the existence of localised ‘hot spots’ of livestock infection during the IEP. Human seropositivity was strongly positively associated with the village-level inter-epidemic RVFV FOI in livestock and a reported history of raw milk consumption. The evidence we find for circulation of RVFV among livestock during the IEP and the very strong association observed between levels of livestock RVFV transmission and human seropositivity, as well as demonstration of raw milk consumption as a risk factor for human infection, highlights the potential risks for zoonotic RVFV spillover in the period between major epidemics in northern Tanzania.

This study provides the first estimates of RVFV infection incidence in northern Tanzania during the IEP. In support of several other studies demonstrating infections during the IEP [11,14,16,53–55], we find clear evidence for the circulation of RVFV among livestock in Tanzania since the last major outbreak in the country in 2007 and 2008. The overall seroprevalence of RVFV infection in livestock from northern Tanzania was considerably lower than has been reported from livestock in other parts of the country sampled over the same time period and tested with the same assay (e.g., 27% in Morogoro Region [11]; 26 and 33% in Mbeya Region [11,16]). The low seroprevalence observed among animals born since the last epidemic translated into a moderately low overall livestock infection incidence of 72 RVFV infections per 10,000 livestock per year across northern Tanzania in the period between 2009 and 2015. Given the differences observed in seroprevalence, it is likely that other parts of the country have substantially higher RVFV transmission intensities during the IEP than we estimate for northern Tanzania. Northern Tanzania has traditionally been considered to be at particularly high risk for RVF, and has been the epicentre of previous outbreaks in the country [4]. The relationship between the intensity of RVFV transmission during the IEP and outbreak risk is poorly understood [56] but our large sample of over 9,000 animals from 43 villages across the region suggest the vast majority of livestock in northern Tanzania have not been exposed to the virus and therefore remain susceptible to infection. This low level of immunity among livestock could be expected to increase the likelihood of large-scale RVF outbreaks when future environmental conditions, such as large scale flooding, allow for large increases in floodwater *Aedes* and *Culex* mosquito populations [1,9].

Despite an overall low seroprevalence, we identified important heterogeneity in livestock RVFV FOI at the village-level. Other studies have reported between-village variation in RVFV livestock infection prevalence and incidence in endemic settings [24,57]. The absence of evidence for spatial autocorrelation in FOI suggests that large-scale, landscape-level effects are less important in explaining heterogeneity in RVFV transmission intensity during the IEP in northern Tanzania than more localised effects operating at smaller spatial scales (i.e., at the village-level). These effects might include local hydrological characteristics, such as the presence of areas at particular risk of flooding, patches of wildlife habitat that may support unknown sylvatic cycles, or small-scale differences in land-use such as the use of irrigated agriculture [58]. Such effects can be expected to influence local mosquito population dynamics, RVFV infection prevalence in mosquitoes, and contact rates between infected mosquitoes and livestock, and therefore RVFV transmission dynamics in livestock [8–10]. In this study we did not evaluate predictors of livestock infection during the IEP, but the between-village variation observed in northern Tanzania suggests this would be a valuable focus for future work.

Previous studies have demonstrated a strong association between human RVFV seropositivity and the presence of RVFV seropositive animals in a household [57]. Here, we find that the average annual village-level force of RVFV infection in livestock (together with individual age and sex) during the IEP explained more than 50% of the between village-heterogeneity in the risk of human RVFV seropositivity. Although there were no RVFV seropositives identified among the small number of people in our sample born since the last epidemic in Tanzania, studies from other settings have clearly demonstrated that human RVFV infections can occur during the IEP in endemic areas [1,59]. The strong positive relationship observed between village-level livestock RVFV transmission intensity during the IEP and human seropositivity in northern Tanzania could therefore be taken to provide strong support to the hypothesis of zoonotic spillover during the IEP across this region. A recent cross-sectional study from three villages in one district of Kilimanjaro Region reporting PCR-based RVFV detection in humans supports this [55]. It is important to note, however, that an alternative explanation for the observed relationship between livestock RVFV FOI during the IEP and human seropositivity is that the villages in which RVFV transmission among livestock was found to be relatively more intense from 2009 through 2015 were the same villages in which high levels of livestock and human infection also occurred during the 2007/08 RVF epidemic. The human seropositives we identify may therefore have been infected during this previous epidemic rather than during the IEP. Antibodies have been detected more than a decade after infection in people [60,61], so this alternative explanation is feasible. Although our age data were limited by broad categorisation, we also did not find an association between a linear increase in 20 year age categories and human seropositivity, a relationship that might be expected if the human exposures (i.e., seropositivity) we observed were principally due to endemic (rather than epidemic) RVFV circulation. Given the feasibility of this alternative explanation, additional studies that can incorporate control for the confounding effect of human exposure during previous epidemics and which can quantify human RVFV infection incidence during the IEP would be valuable to confirm zoonotic RVFV spillover during the IEP across northern Tanzania. It is noteworthy that if this proposed alternative explanation was found to be correct (i.e., that the relationship we observed between livestock RVFV FOI during the IEP and human seropositivity was confounded by human infection during previous epidemics), it would suggest similar risk factors for livestock RVFV infection during the IEP and epidemic periods. This is an an area that has had relatively limited exploration [56]. It would also suggest communities with the highest levels of RVFV transmission among livestock during the IEP are likely to be the communities in which the largest number of human infections during outbreaks could be expected.

We found evidence for a strong positive association between the reported history of raw milk consumption and human RVFV seropositivity, including in the model with penalisation for the number of co-efficients evaluated. This supports recent work by Grossi-Soyster et al [62] who analysed relationships between raw milk consumption and human RVFV infection risk in detail. An earlier systematic review and meta-analysis also confirmed consumption of raw milk as a risk factor for human infection both during outbreaks and the IEP [63]. Little is known about the likelihood or duration of RVFV shedding in the milk of infected animals, or survival of RVFV in milk, but recent work by our group detected RVFV nucleic acids in the milk of recently aborted cattle in northern Tanzania [6]. In Tanzania, milk can be transported over long distances through trade, including to urban populations who may have minimal direct contact with livestock [64]. Hence, while our data show that living in a village in which RVFV circulates at high levels is an important predictor of human seropositivity, any people who consume raw milk products from such villages may also be at elevated risk for infection with RVFV. Urban residence or lack of direct contact with livestock should therefore not necessarily rule out RVF as a differential diagnosis for undifferentiated fever in people.

There are several limitations that should be considered when interpreting our estimates of RVFV seroprevalence, FOI and infection incidence. Given the low overall seroprevalence of RVFV observed, our estimates are strongly dependent on the assumption of very high specificity of the commercial ELISA used to identify seropositive individuals. Published test performance estimates suggest that this test has a very low false positivity ratio (33,47), but it is important to note that even a small decrease in the test specificity resulted in substantial reductions in the estimated FOI (and therefore infection incidence). Future studies could significantly reduce the potential for this bias by confirming ELISA-based livestock seropositives with the highly specific PRNT, which was not possible in our study due to resource constraints. Other assumptions include the expectation that antibody levels following infection remain detectable over the course of an animal’s life. There have been only a limited number of studies to explore RVFV antibody kinetics in livestock, but detectable RVFV IgG antibodies are considered to last for several years [29].

The general FOI models described here also assumed no infection-related mortality. This is not the case for infection with RVFV, for which mortality in susceptible animals, particularly among neonates, can be high [65]. This is an important violation of the assumptions of this model that means FOI, and therefore infection incidence, may have been underestimated to an unknown extent. Although our estimates of disease incidence should be interpreted in the light of this limitation, a similar consideration applies whenever seroprevalence of RVFV among animals born during the IEP is taken to provide an indication of the levels of RVFV infection during this period. Differences in estimated FOI and incidence (as well as seroprevalence) between species, which are known to have different susceptibility to RVF-associated mortality [65], are particularly noteworthy in this context. The FOI for cattle and sheep were broadly equivalent but this was considerably lower in goats. One potential explanation for this finding is that goats are less attractive to mosquito vectors of RVFV than either sheep or cattle, and therefore less likely to be infected. There have been few studies to evaluate host biting preferences for potential RVFV vectors in endemic settings. A recent study in Kenya suggested that while cattle were the most attractive livestock species to *Culex* mosquitoes, there was no difference in attractiveness between sheep and goats, suggesting both may have similar risk of mosquito-borne RVFV infection [66]. An alternative explanation is that goats suffer from higher infection-related mortality than either sheep or cattle. This does not reflect experimental evidence, which tends to suggest cattle are most resistant to RVFV infection, followed by goats, then sheep, which are widely thought to suffer the highest levels of clinical disease and mortality of all ruminant species [65]. However, in addition to this species effect, it has been widely noted that clinical and pathological outcomes can be strongly influenced by the breed of host animals [67,68]. Almost all sheep in the study area are of the Red Maasai breed or their crosses [69], which are endemic to the Rift Valley area. The Red Maasai breed is highly valued by pastoralists and agro-pastoralists in Kenya and Tanzania for its resistance to local pathogens [70–72], and it is conceivable that this breed also has higher RVF resistance than goats and other breeds of sheep. Better understanding of the susceptibility of local breeds of livestock to RVF in endemic areas would be valuable for understanding both the impacts and epidemiology of RVF outbreaks in Tanzania, particularly since the modernisation of the livestock sector, including the introduction of more productive ‘exotic’ livestock breeds, is strongly promoted in the country [73]. Given the low overall RVFV seroprevalence (and therefore low FOI) in livestock, and particularly low seroprevalence among goats, as well as our relatively small human sample size, we did not evaluate the relative contribution of the inter-epidemic force of RVFV infection in different livestock species on human seropositivity. This question has clear relevance for the design of veterinary public health interventions.

## Conclusion

Using a general FOI approach, we estimated the average annual population-level RVFV infection incidence among cattle, goats and sheep across northern Tanzania in the period since the last reported epidemic in the country. Our results provide the first population-level estimates for RVFV infection incidence for this region that has been the epicentre of past RVF epidemics in Tanzania. We show that RVFV has circulated at apparently low levels among livestock during the IEP but that important levels of heterogeneity in transmission intensity occured at the village-level. The vast majority of cattle, goats, and sheep across the region were unexposed to RVFV and therefore susceptible to infection in the event of environmental conditions that would support rapid increases in transmission of the virus. We found a strong positive association between the village-level RVFV FOI livestock and risk of human RVFV seropositivity. These findings provide support to the hypothesis of undetected zoonotic spillover associated with inter-epidemic circulation of RVFV among livestock in northern Tanzania, as has been described in other endemic areas. We also find evidence that raw milk consumption is strongly associated with human seropositivity in northern Tanzania, supporting previous findings that highlighted a role for raw milk consumption in zoonotic RVFV transmission. Our study demonstrates the value of applying a general force of infection approach to serosurvey data that can allow quantification of historic RVFV infection incidence during period in which no RVF cases were reported among people or animals.

## Supporting information

S1 File Supplementary Materials. (DOCX)
