# Supplementary material for: Inter-epidemic Rift Valley fever virus infection incidence and risks for zoonotic spillover in northern Tanzania
Source: PLoS Negl Trop Dis. 2022 Oct 28;16(10):e0010871. doi: 10.1371/journal.pntd.0010871 (PMC9665400; doi:10.1371/journal.pntd.0010871)
Supplement: S1 File — (DOCX) [file pntd.0010871.s001.docx]

**Supplementary Information for:**

**Inter-epidemic Rift Valley fever virus infection in livestock and risks for zoonotic spillover in northern Tanzania**

Corresponding authors:

William A. de Glanville ([will.deglanville@glasgow.ac.uk](mailto:will.deglanville@glasgow.ac.uk))

Sarah Cleaveland ([sarah.cleaveland@glasgow.ac.uk](mailto:sarah.cleaveland@glasgow.ac.uk))

**S.1. Further details on serological assays**

**S.1.1. Glasgow in-house ELISA**

For the University of Glasgow in-house ELISA procedure, plates were coated with 100 µl diluted Rift Valley fever virus (RVFV)-N protein and incubated at 4 °C overnight then washed three times with wash buffer. 200 µl of blocking buffer was added to each well and incubated for one hour at room temperature. Plates were washed three times and 100 µl diluted positive control sera, negative control sera and 100μl of test sera added in duplicate, covered and incubated at room temperature for 2 hours. 100 µl of horseradish peroxidase (HRP)-conjugated anti-species (goat or sheep) antibody (Thermo Fisher Scientific, ‎[Massachusetts](https://en.wikipedia.org/wiki/Massachusetts), USA) diluted to 1:1000 was added to each well, covered and incubated at room temperature for one hour. After incubation, plates were washed five times with wash buffer and 100μl of TMB (3,3',5,5'-tetramethylbenzidine) substrate was added to each well and the plates kept in the dark for 15 minutes to allow colour change. 100 µl of stop solution (2 N H_2_SO_4_) was added per well and the absorbance read at 650nm. Optical density (OD) values were expressed as a percentage positive of the positive control. The RVFV-N protein was generously prepared by Dr. Ping Li, University of Glasgow.

A total of 2414 SEEDZ goat samples and 2053 SEEDZ sheep samples were tested with the in-house assay. Of the goats, 758 (31%) were positive on the in-house assay. Of the sheep, 620 (30%) were positive on the in-house assay. In addition to all of the 758 in-house ELISA positive goat samples, 330 (20%) randomly selected in-house assay negative goats were also tested with the IDVet commercial cELISA. In addition to all of the 620 in-house ELISA positive sheep samples, 257 (18%) randomly selected in-house assay negative sheep were also tested with the IDVet commercial cELISA. Two-by-two tables with the species-specific results of these comparisons are shown in Table S1 and S3. The overall percent positive agreement (for both species combined, i.e., 86/88) was high at 98% (95% CI 91 – 100), suggesting the use of the in-house ELISA as a screening test is unlikely to have resulted in a substantial number of missed seropositive results among the SEEDZ sheep and goats. The overall percent negative agreement (585/1877) was very low (31% (95% CI 29 – 33). However, given that all in-house positives were confirmed with the IDVet assay, this is not expected to have impacted on our findings.

It is important to note that the total fraction of the in-house ELISA test negative samples tested with the commercial cELISA ((330+257)/(1656+1433), or 19%) is considerably less than the fraction of in-house ELISA test positive samples (100%). This reduces our ability to observe false negative results, adding additional uncertainty to the observed percent positive agreement (i.e., the numerator in the percent positive agreement calculation can be considered fully observed since all in-house ELISA test positives were retested while the denominator is not fully observed since only a proportion of in-house ELISA test negatives were retested) [1]. For example, weighting the observed number of false negatives by a testing fraction of 19% to replicate what we might expect in the scenario in which all in-house ELISA test negatives were retested with the commercial cELISA would result in a reduced estimated percent positive agreement of around 90% (i.e., 86/(86 + (2/0.19)). The same logic could be applied to the observed percent negative agreement, where false positives are fully observed but the unequal sampling fraction means we have reduced opportunity to observe true negatives. Hence, weighting to provide adjustment for the number of true negatives we might expect to observe if all in-house ELISA negatives were also tested with the commercial cELISA would result in a higher estimated negative percent agreement of around 60% (i.e., (585/0.19)/((585/0.19) + 1877)).

**Table S1**. Two-by-two table showing comparison between results of the Glasgow in-house ELISA assay with the commercial IDVet cELISA assay for goat samples collected through the SEEDZ study in northern Tanzania.

|  | In house + | In house - | Total |
| --- | --- | --- | --- |
| IDVet + | 28 | 1 | 29 |
| IDVet - | 730 | 329 | 1059 |
| Total | 758 | 330 | 1088 |

**Table S2**. Two-by-two table showing comparison between results of the Glasgow in-house ELISA assay with the commercial IDVet cELISA assay for sheep samples collected through the SEEDZ study in northern Tanzania.

|  | In house + | In house - | Total |
| --- | --- | --- | --- |
| IDVet + | 58 | 1 | 59 |
| IDVet - | 562 | 256 | 818 |
| Total | 620 | 257 | 877 |

**S.1.2. Plaque Reduction Neutralisation Test (PRNT)**

Recombinant live attenuated MP12 strain of RVFV [2] was used. The overlay was prepared by mixing 1.2% Avicel solution with 2XMEM (minimum essential medium, Gibco) containing 4% FCS (foetal calf serum) v/v) in 1:1 ratio. 2X MEM was made by combining 20% (v/v) 10x Modified Eagle’s Medium (MEM) (Gibco), 2% (v/v) L-glutamine, 0.435% (v/v) NaHCO3, diluted in distilled water. Crystal Violet stain was prepared by mixing 10 ml Methanol and 200 ml Ethanol absolute and 1 g Methyl Violet (Crystal Violet), mixed well by shaking to dissolve the methyl violet. 100 ml formaldehyde solution (41%) was added followed by distilled water up to a volume of 1L.

The PRNT was performed using Vero E6 cells, seeded a day prior to infection in 12 well plates at a cell density of 1.5 x 10^5^ cells per well. The next day, 4-fold dilutions (from 1/32 to 1/32768) of the sera samples were prepared in a 96 well plate in DMEM (Dulbecco's modified eagle medium, Gibco) supplemented with 2% FCS (150ul per well). Then 150ul of DMEM containing 100 pfu of rMP-12 virus was added to diluted sera into each well and plates were incubated at 37 °C for 1h. Next, the media was removed from the cells. Thereafter, 200ul of each mixture of serum and MP-12 virus was added to infect confluent monolayers of Vero E6 cells in 12-well plates. After 1h incubation at 37 °C the supernatant was removed, then 1ml overlay (0.6% Avicel, 2x MEM, 2% FCS) was added per well, incubated at 37 °C for 4 days, after which the cells were fixed with 1ml of 8% formaldehyde in PBS (v/v) per well and incubated for at least 1h at room temperature. Formaldehyde and avicel overlays were removed and plates washed in water, then stained with crystal violet stain followed by washing the plates with water and then left to dry. The plaques were then counted. Counts from replicate wells were averaged, and the average was multiplied by the dilution factor of the inoculum, which produced that number, and the volume of inoculum plated to calculate the plaque forming units (PFU) per mL of the original stock virus preparation. The PRNT was performed under biosafety level 3 (BSL-3) conditions at the University of Glasgow.

Comparison of PRNT results with the 26 positive competitive ELISA results is shown in Table S3. The cut-off for the ELISA was <40 and for the PRNT >32.

**Table S3. Comparison of PRNT and ELISA results for 26 human ELISA positive samples.**

| **PRNT (90%)** | **ELISA SN** |
| --- | --- |
| 2580.5 | 10.8 |
| 2246.8 | 3.6 |
| 1913.6 | 9.9 |
| 1817.6 | 4.4 |
| 1638.4 | 5.7 |
| 1303.6 | 4.9 |
| 1244.6 | 5.8 |
| 1089.8 | 5.6 |
| 1030.4 | 13.1 |
| 972.8 | 8.9 |
| 907.0 | 3.4 |
| 896.0 | 11.1 |
| 843.5 | 27.9 |
| 651.9 | 11.3 |
| 646.4 | 5.8 |
| 535.4 | 10.3 |
| 512.0 | 17.7 |
| 347.9 | 20.9 |
| 286.8 | 12.9 |
| 261.5 | 10.7 |
| 251.7 | 39.0 |
| 251.4 | 7.8 |
| 238.9 | 10.6 |
| 232.7 | 16.6 |
| 224.0 | 5.8 |
| 149.9 | 6.4 |

**S.2. Livestock ageing**

Literature based estimates for eruption times of permanent incisors were used to age cattle [3], goats [4], and sheep [4,5]. The ages in years assigned for each species are shown in Table S4.

**Table S4.** Animal ages assigned on the basis of dentition

| **Dentition category** | **Cattle age (years)** | **Goat age (years)** | **Sheep age (years)** |
| --- | --- | --- | --- |
| Temporary teeth | 1.0 | 0.6 | 0.75 |
| One pair incisor | 2.0 | 1.4 | 1.6 |
| Two pair incisors | 3.0 | 1.8 | 2.4 |
| Three pair incisors | 4.0 | 2.3 | 3.0 |
| Four pair incisors | 7.3 | 5.2 | 5.3 |
| Four pair and worn | 9.9 | 6.6 | 6.1 |

**S.3. Estimation of the force of infection**

Models were implemented in JAGS from within R [6].

The following model specification in JAGS represents Equation 1 in the main text (text/values following # given for information):

model{

for (i in 1:n) { # n = number of individuals

rvf[i] ~ dbern(p[i])

p[i] <- 1 - exp(-l*age[i])

}

# priors

l ~ dunif(0,1)

}

As described in the main text, this is equivalent to a binomial GLM with a clog-log link:

model{

for (i in 1:n) { # n = number of individuals

rvf[i] ~ dbern(p[i])

cloglog(p[i]) <- bcons + 1*log(age[i])

}

# priors

bcons ~ dnorm(0, 1.0E-06)

}

Either model can be extended to account for diagnostic specificity (Equation 3 in the main text):

model{

for (i in 1:n) {

rvf[i] ~ dbern(pa[i])

pa[i] <- p[i]*se+(1-p[i])*(1-sp)

p[i] <- 1 - exp(-l*age[i])

}

# priors

l ~ dunif(0,1)

se <- 1

sp <- 0.99 # values ranging from 1 to 0.9

}

The GLM was also further extended to a GLMM through the inclusion of a village-level random effect:

model{

for (i in 1:n) { # n = number of individuals

rvf[i] ~ dbern(p[i])

cloglog(p[i]) <- 1*log(age[i]) + a[vid[i]]

}

for (k in 1:nn) { # nn = number of villages

v[k] ~ dnorm(0, tau.v)

}

# priors

sigma2.v <- pow(sigma.v, 2)

sigma.v ~ dunif(0, 100)

tau.v <- 1/sigma2.v

mu.v ~ dnorm(0, 1.0E-06)

for (i in 1:nn) {

a[i] ~ dnorm(mu.v,tau.v)

}

}

**References**

1. Dohoo I, Martin W, Stryhn H. Methods in Epidemiologic Research. VER Inc., Charlottetown, Prince Edward Island, Canada; 2012.

2. Caplen H, Peters CJ, Bishop DHL. Mutagen-directed Attenuation of Rift Valley Fever Virus as a Method for Vaccine Development. J Gen Virol. 1985;66: 2271–2277. doi:10.1099/0022-1317-66-10-2271

3. Carles AB, Lampkin KM. Studies of the permanent incisor eruption, and body development, of the Large East African Zebu (Boran): 1. The ages at first appearance of the incisors, lengths of the incisor eruption period, and sources of variation. The Journal of Agricultural Science. 1977;88: 341–360.

4. Wilson RT, Durkin JW. Age at permanent incisor eruption in indigenous goats and sheep in semi-arid Africa. Livestock Production Science. 1984;11: 451–455.

5. Cocquyt G, Driessen B, Simoens P. Variability in the eruption of the permanent incisor teeth in sheep. Vet Rec. 2005;157: 619–623. doi:10.1136/vr.157.20.619

6. Su Y, Yajima M. R2jags: Using R to Run “JAGS”. R package version 0.5-7. 2015 Aug.
